# Occupational Differences in Metabolic Syndrome Incidence Among Older Workers

**DOI:** 10.1093/geroni/igab046.198

**Published:** 2021-12-17

**Authors:** Katharina Runge, Sander K R van Zon, Ute Bültmann, Kène Henkens

**Affiliations:** 1 Netherlands Interdisciplinary Demographic Institute (NIDI), The Hague, Zuid-Holland, Netherlands; 2 University Medical Center Groningen (UMCG), Groningen, Groningen, Netherlands

## Abstract

This study investigates whether the incidence of metabolic syndrome (MetS), and its components, differs by occupational group among older workers (45-65 years) and whether health behaviors (smoking, leisure-time physical activity, diet quality) can explain these differences. We analyzed data from older workers (N=23 051) from two comprehensive measurement waves of the Lifelines Cohort Study and Biobank. MetS components were determined by physical measurements, blood markers, medication use, and self-reports. Occupational group and health behaviors were assessed by questionnaires. The association between occupational groups and MetS incidence was examined using Cox regression analysis. Health behaviors were subsequently added to the model to examine whether they can explain differences in MetS incidence between occupational groups. Low skilled white-collar (HR: 1.25, 95% CI: 1.13, 1.39) and low skilled blue-collar (HR: 1.45, 95% CI: 1.25, 1.69) workers had a significantly higher MetS incidence risk during 3.65 years follow-up than high skilled white-collar workers. Health behaviors reduced the strength of the association between occupational group and MetS incidence most among low skilled blue-collar workers (i.e. 10.3% reduction) as unhealthy behaviors were more prevalent in this occupational group. Similar occupational differences were observed on MetS component level. To conclude, MetS incidence in older workers differs between occupational groups and health behaviors only explain a small part of these differences. Health promotion tailored to occupational groups may be beneficial specifically among older low skilled blue-collar workers. Research into other factors that contribute to occupational differences is needed, as well as studies spanning the entire working life course.

